# “It’s hot potato, nobody wants these people”: Palliative and end-of-life experiences among people with opioid use disorder and life-limiting illness

**DOI:** 10.1177/26323524261492741

**Published:** 2026-09-28

**Authors:** Lisa M. Boucher, Jenny Lau, Rebecca Bagnarol, Kyle Drouillard, Ladees Al Hafi, Sheila Jennings, Andrew McLeod, Camilla Zimmermann, Teighan Killackey, Joanna Binch, Anne Meaghen Hagarty, Taliesin Cahill, Nicole Buchanan, Tara Gomes, Sarina R. Isenberg

**Affiliations:** 1 Bruyère Health Research Institute, Ottawa, ON, Canada; 2 Department of Medicine, Faculty of Medicine, 6363 University of Ottawa, Ottawa, ON, Canada; 3 Department of Supportive Care, Princess Margaret Cancer Centre, 7989 University Health Network, Toronto, ON, Canada; 4 Division of Palliative Medicine, Department of Medicine, 70379 University of Toronto, Toronto, ON, Canada; 5 Legal Studies Department, Ontario Tech University, Oshawa, ON, Canada; 6 Disability Studies Program, Toronto Metropolitan University, Toronto, ON, Canada; 7 Alpha House Recovery Community, Toronto, ON, Canada; 8 META:PHI, Toronto, ON, Canada; 9 Lawrence Bloomberg Faculty of Nursing, 70379 University of Toronto, Toronto, ON, Canada; 10 Toronto General Hospital Research Institute, Toronto, ON, Canada; 11 Sandy Hill Community Health Centre, Ottawa, ON, Canada; 12 27337 The Ottawa Hospital, Ottawa, ON, Canada; 13 Palliative Education and Care for the Homeless (PEACH), 585182 Inner City Health Associates, Toronto, ON, Canada; 14 Trillium Health Partners, Mississauga, ON, Canada; 15 Department of Family and Community Medicine, 70379 University of Toronto, Toronto, ON, Canada; 16 Leslie Dan Faculty of Pharmacy, 70379 University of Toronto, Toronto, ON, Canada

**Keywords:** opioid use disorder, palliative care, end of life, health inequities, stigma, interdisciplinarity, trauma-informed care, harm reduction, community-based services, qualitative research

## Abstract

**Background:**

People with opioid use disorder (OUD) experience disproportionate premature mortality compared to the general population, yet receive less palliative care. End-of-life experiences of people with OUD and life-limiting illness, and of healthcare providers caring for these individuals, remain poorly understood.

**Objectives:**

To explore how people with OUD access and experience palliative and end-of-life care, and how providers navigate caring for these patients.

**Design:**

The qualitative phase of a mixed methods study using applied thematic analysis informed by stigma theory.

**Methods:**

We recruited participants across Canada using purposive and snowball sampling. Participants included 19 people with OUD and life-limiting illness (whether or not they received palliative care) or their caregivers, and 44 healthcare providers across diverse settings. We collected data using semi-structured interviews and focus groups. We coded transcripts via a codebook of inductively- and deductively-developed codes, and distilled themes through iterative team synthesis.

**Results:**

People with OUD faced pervasive barriers accessing quality palliative and end-of-life care, including three themes of complex health and social needs, stigma/discrimination in care, and broken healthcare systems. These challenges led to missed diagnoses, limited palliative care, reluctance to enter inpatient settings, and difficulty with prognostication, often resulting in negative outcomes. Conversely, the few patients with OUD who received specialized palliative care mostly cited positive experiences. A fourth theme centers on providers considering interdisciplinary collaboration to be both a barrier and solution. The final theme highlights other solutions, including innovative, tailored supports, comprising trauma-informed and harm reduction approaches and unique community-based services.

**Conclusion:**

Palliative care systems should further embrace and integrate patient-centered care for people with OUD, which includes trauma-informed and harm reduction approaches, with greater investment in innovative community-based services. Improved training and collaboration are also required for palliative care and addiction medicine/harm reduction providers to ensure patients with OUD receive sufficient care across settings.

## Plain language summary

People with opioid use disorder die at younger ages than people in the general population, but they receive less healthcare when diagnosed with a life-threatening illness. We aimed to better understand their experiences near the end of life, including how healthcare providers manage caring for these patients. We conducted a qualitative study by recruiting participants across many different settings and leading interviews and focus groups to discuss their experiences. Participants included people with opioid use disorder and life-threatening illness (whether or not they received specialized palliative care) or their family or friend caregivers, and their healthcare providers. We analysed and coded the data carefully and interpreted it through discussion with our diverse team. People with opioid use disorder faced many barriers accessing quality care near the end of life, including complex health and social needs, stigma and discrimination in care, and broken healthcare systems. These challenges led to missed diagnoses, limited palliative care, reluctance to enter hospitals or hospices, and difficulty predicting when patients would die. On the other hand, the few patients with opioid use disorder who received palliative care mostly reported positive experiences. Providers considered collaborating across disciplines to be both a barrier and solution. Other solutions included inventing new supports or tailoring supports to patients’ needs, including through community-based services and approaches intended to reduce substance use harm or address how prior trauma shapes current experiences. Palliative care systems should embrace trauma-informed and harm reduction approaches for people with opioid use disorder, and invest more in unique community-based services. Improved training and collaboration are required for mainstream palliative care providers and addiction medicine providers to ensure patients with opioid use disorder receive sufficient care across settings.

## Introduction

People with opioid use disorder (OUD) face disproportionately high risks of premature death, both from opioid toxicity and other life-limiting conditions.^1–3^ Yet, Canadian health administrative data indicate that people with OUD receive less palliative care overall, including reduced access to community-based palliative services in their final months of life.^3,4^ Across international literature, palliative responses to substance use tend to be fragmented and oriented toward risk management rather than patient needs and preferences. A rapid review (2018-2023) found that much of the research focuses on monitoring, prescribing policies, or medication management, rather than the structural or relational barriers facing people with OUD near the end of life.^5^ Studies in the United States, Germany and Australia highlighted palliative providers’ perceived unpreparedness and use of inconsistent approaches to caring for people with OUD or another substance use disorder.^6–8^

Multiple barriers may contribute to these inequities. Providers report insufficient training in addiction medicine and palliative care, limited access to specialist expertise, and heavy workloads that constrain time for advance care planning and complex care coordination.^9^ People with OUD also face broad structural vulnerabilities related to poverty, housing instability, and criminalization that complicate serious illness care,^10–13^ as well as discrimination in healthcare settings.^14,15^ While these barriers have been documented in palliative care for similar patient groups (e.g., those who use other substances or experience homelessness),^13,16–18^ their specific relevance for people with OUD and life-limiting illness remains poorly understood.

Together, these gaps highlight limitations in existing evidence. Although recent research using health administrative databases provides valuable system-level insights on palliative care utilization among people with OUD in our setting, these data sources cannot speak to the experiences of patients, caregivers, or healthcare providers,^3,4^ underscoring the need for complementary qualitative research.^19^ Thus, our study aims to explore how people with OUD access, encounter, or are excluded from palliative and end-of-life care, including those living in poverty and experiencing homelessness. We examine how social or structural determinants of health, including stigmatization, shape these experiences, and how providers perceive and navigate the challenges of caring for these patients near the end of life.

## Methods

### Study design

The study was part of a larger mixed methods explanatory design,^20^ but this article reports on the qualitative phase only. Compatible with mixed methods studies, we used a qualitative descriptive approach to explore the perspectives of patients/caregivers and healthcare providers on end-of-life experiences including palliative care among people with OUD.^21,22^ We recruited participants in-person in Ottawa and Toronto, Ontario, and virtually across Canada, from November 2023 to April 2025. We received research ethics board approval from Bruyère Health, The Ottawa Hospital, and University Health Network.

### Theoretical framework

Given that people who use drugs are highly stigmatized and stigma is a known barrier affecting their healthcare access,^23–26^ we anticipated that stigma and discrimination might also affect their palliative care and end-of-life experiences. There are three levels of stigma that we applied in this study: 1) structural, 2) social, and 3) self-stigma. Structural stigma is evoked through institutional policies and permeates workplace culture, such as zero-tolerance for drug use on hospital property.^23,27^ Social stigma is evident in the stereotypes and prejudices that the public and healthcare providers have toward drug use, affecting how these patients are treated.^23,26^ Self-stigma involves internalizing social stigma, resulting in low self-esteem and avoidance of healthcare.^24,28^ We used this conceptualization to develop several interview questions and to frame the deductive part of our analysis.

### Sampling and recruitment

We utilized purposive and snowball sampling strategies.^29^ In collaboration with healthcare institutions, we invited individuals with OUD receiving specialized palliative care, or their caregivers, to participate in interviews. We engaged participants not accessing palliative care, or their caregivers, via community-based organizations and patient or caregiver advocacy networks. We aimed to reach diverse patients, accounting for gender, age, ethnicity, Indigeneity, socioeconomic status, geography, comorbidities, and healthcare setting. Participants were excluded if they did not have a life-limiting illness or did not report an opioid-related health experience indicative of OUD within the past three years.

We recruited healthcare providers from clinics, hospitals, hospices, and community-based organizations to participate in focus groups or interviews. We primarily recruited palliative care and addiction medicine physicians and nurses, though also included hospitalist and primary care providers. We also invited social workers, pharmacists, harm reduction workers and other professionals who had provided palliative care to patients with OUD. We use the term mainstream palliative care in this article as it was used by study participants to refer to healthcare providers who primarily provide palliative care for the general population, often in traditional settings.

### Participant characteristics

Nineteen patients/caregivers and 44 healthcare providers participated (n=63) within 44 interviews and three focus groups. The average length was 47 minutes for interviews and 67 minutes for focus groups. Table 1 and Table 2 provide demographic and clinical/practice characteristics for patient and provider participants, respectively.

**Table 1.** Demographic and clinical characteristics of patient participants (n=17)^A^.

Characteristic	N (%)
Gender	
Man	11 (64.7)
Woman	5 (29.4)
Non-binary	1 (5.9)
Age	
18-44	4 (23.5)
45-54	4 (23.5)
55-64	4 (23.5)
65-74	3 (17.6)
75-84	2 (11.8)
Race/Ethnicity	
Indigenous	3 (17.6)
Visible Minority	1 (5.9)
Neither	13 (76.5)
Rurality	
Urban	16 (94.1)
Rural	1 (5.9)
Opioid-related Health Experiences Indicative of Opioid Use Disorder^B^	
Received Opioid Agonist Therapy	12 (70.6)
Visited Emergency Department	9 (52.9)
Hospitalized	5 (29.4)
Other^C^	4 (23.5)
Palliative Care Received	
Yes^D^	4 (23.5)
No	13 (76.5)

^A^The total here does not equal the total number of patient/caregiver participants (n=19), as two caregivers reported providing care for multiple patients.

^B^Participants could select more than one response.

^C^Other self-reported responses included: overdosing within the community, receiving safer supply, or misuse of opioid prescriptions.

^D^Receipt of palliative care in distinct settings: one each in a hospital, hospice, clinic, and palliative care unit.

**Table 2.** Demographic and practice characteristics of healthcare provider participants (n=44).

Characteristic	N (%)
Gender^A^	
Man	12 (25.6)
Woman	30 (67.4)
Non-binary	1 (2.3)
Role	
Physician	20 (45.5)
Nurse^B^	13 (29.5)
Social Worker	1 (2.3)
Pharmacist	1 (2.3)
Community Worker	2 (4.5)
Other^C^	7 (15.9)
Years in Practice, *mean (range)*	12.6 (1-40)
Location of Practice^D^	
Hospital	21 (48.8)
Clinic	14 (32.6)
Home	12 (27.9)
Hospice	11 (25.6)
Shelter/Supportive Housing	5 (11.6)
Community	4 (9.3)
Palliative Care Unit	1 (2.3)
Pharmacy	1 (2.3)
First Nations Community	1 (2.3)
Patients with OUD in Practice, *% (range)*^E^	35.7 (1-100)
Rurality^F^	
Urban	43 (97.7)
Rural	1 (2.3)
Training in Palliative Care^G^	
Yes	36 (81.8)
No	8 (18.2)

^A^One provider responded Prefer not to answer.

^B^Roles include advanced practice nurse, nurse practitioner, clinical nurse specialist, palliative care nurse specialist, nurse coordinator, and registered nurse.

^C^Includes one of each: psychiatrist, clinical coach, paramedic, Indigenous patient navigator, personal support worker, client care worker, and hospice manager.

^D^Participants could select more than one response.

^E^These data are approximate, as some respondents reported ranges rather than exact values (we used the medians to calculate the overall mean percentage here).

^F^Some providers also delivered care in rural settings; however, they were only able to select one response.

^G^Those who reported any training in palliative care described receiving it through formal education, residency, fellowship, added competency, workplace training, or specific programs such as Pallium Canada’s LEAP course.

There were 11 patients, six caregivers, and two others who identified as both patient and caregiver. Caregivers reported being a partner, sibling, friend, or chosen family member to the patient with OUD and life-limiting illness for whom they cared, and they provided the sociodemographic responses for these patients. Four patients received palliative care, 11 were men, most lived in urban areas, and most reported recent receipt of opioid agonist therapy for OUD treatment or having an emergency department visit or hospitalization because of opioid use.

Providers were largely women nurses or physicians who reported an average of 13 years in practice (with a range of 1 to 40 years), across diverse healthcare settings that were mostly in urban areas. Most reported receiving some palliative care training, and their percentage of patients who had OUD ranged from 1% to 100%.

### Data collection

After obtaining written or verbal consent, we held interviews virtually or in-person, depending on participant preference and feasibility, at convenient community or clinical sites, or via videoconference or telephone. We conducted in-person focus groups with staff at community organizations that had mandates to support the health needs of marginalized populations. Separate semi-structured interview/focus group question guides were developed to guide data collection with each participant group, including two guides for patients/caregivers (those receiving vs. not receiving specialized palliative care) and one guide for healthcare providers, to capture their perspectives on patients’ end-of-life experiences. Brief sociodemographic surveys for patients and providers were also developed and administered by the research team (see Supplementary Material). Data collection was led by a postdoctoral fellow with 10 years of experience in community-based research with people who use drugs, though some interviews were conducted by other staff members after they received training. Participants received a $50 CAD honorarium or gift card for their time.

The interviews and focus groups were recorded and transcribed verbatim by a contracted transcriptionist, then verified against the recording and deidentified by members of our research team. Participants were assigned code numbers during data collection; however, to support readers’ interpretation, with the quotations we include descriptive labels indicating each participant’s role (patient, caregiver, or type of healthcare provider) and, if applicable, the setting(s) in which they provided care.

### Data analysis

Data analysis followed an applied thematic approach.^30,31^ First, seven team members hand coded several distinct transcripts and developed a codebook through combining inductive coding and deductive concepts derived from stigma theory.^23,27,28^ Then, using MAXQDA software,^32^ four members group coded one transcript, followed by iterative consensus coding of three transcripts, for which we independently coded before merging individual transcripts into a combined file. We reviewed this file for consistency, resolving discrepancies and establishing agreement through discussion and intersubjective assessment.^31^ Subsequently, two team members independently coded all remaining transcripts. Finally, two members summarized the coded segments, then synthesized summaries into a thematic structure through discussions with our diverse team of researchers, healthcare providers, and people with lived expertise. Data collection and analysis occurred iteratively, and after identifying no new codes or themes in the last three interviews, we confirmed that thematic saturation was achieved.^33^

We improved rigour in our study by considering trustworthiness criteria and employing numerous techniques.^34–37^ In data collection, we used honesty-boosting strategies such as building rapport and iterative questioning, and included many types of informants and sites to support triangulation. In analysis, we used line-by-line coding and constant comparison, examined our social positions and potential biases, documented challenges and decisions, and checked our interpretation with diverse interest holders. In reporting, we include thick description to support readers’ own evaluation of the evidence, and provide rich contextual information to facilitate assessment of transferability.

## Results

Five themes were identified in the data. Providers and patients/caregivers described three core challenges that hindered access to quality palliative and end-of-life care for people with OUD and life-limiting illness: 1) complex health and social needs, 2) stigma/discrimination in care, and 3) broken healthcare systems. Additionally, participants highlighted a fourth theme that interdisciplinary care was both a barrier and a solution, while emphasizing a fifth theme about the value of tailored and innovative approaches to care, including patient-centered, trauma-informed, harm reduction, and community-based supports. Table 3 provides additional quotes for all five themes.

**Table 3.** Additional quotes reflecting the themes and sub-themes.

Theme 1: Complex health and social needs	
Sub-themes	Quotes
Structural vulnerability	*“Income and housing, for sure, and leading to, like, food insecurities, right? So, barriers even to transportation of getting to and from appointments, transportation to getting food, transportation to getting any services, that is, like, yeah, I’d say for us that that’s a huge piece.”* (Paramedic, community)
	*“Mobility. A lot of people in wheelchairs. This is the main reason I left [that organizational role] too, was they mostly have these shitty rackety old wheelchairs. And trying to push someone who’s got issues in regards to their weight, pushing around a 300-lb person in a shitty wheelchair that doesn’t work through snowbanks in February. That prevented so much engagement with care, that could just blow my mind. The fact that these poor folks who could barely walk would have to jump on three buses in December to get the care that they needed. A lot of people, for them it’s easier to just say, ‘Fuck it,’ you know*…*”* (Harm reduction worker, home/supportive housing/hospice)
Survival mindset	*“Some of that is the skillset that they’ve learned to survive on the street doesn’t always serve them well in terms of accessing the system. And so, it’s just like, yeah, this has let you survive on the street, this is not going to help you with your end of life, with those symptoms.”* (Physician, community-based clinic)
	*“I don’t have a lot of folks where they’ve got a diagnosis and we’re able to support them and do some advanced care planning with them and have that time. It’s a luxury. It’s not something that I find that we have out here. That’s a more – straight people have that.”* (Caregiver)
	*“Or they’re really actively using, and everything is chaos for them. So, those are sort of the typical barriers. Or they end up in an emergency room, instead of the appointment they were supposed to get to, that would help them connect with palliative care. So, they live from crisis to crisis. And that’s when they access care.”* (Physician, hospital/home)
	*“On average, it seems greater to us that people have never really given much contemplation about who they want to have contacted, do they want to try to reconcile with past people in their lives, where should their body go, should they be cremated, all these sorts of different things. And that seems to happen at a higher level with opioids because of the fact that a severe opioid use disorder and opioid-related harms is so all-consuming for their day-to-day life for so many years that they never get to think about it and/or the spontaneity in which it comes out.”* (Physician, hospital/home/shelter)
	*“So that whole thing of people going out and getting their own drugs, right, because that whole piece of going from controlling your own drug supply to—and they may be on safe supply but also still using fentanyl—to then getting bedbound and relying on us solely to take their pain away. That’s a really hard transition for people on so many levels and requires lot of trust in your healthcare provider.”* (Nurse, community-based hospice)
Multimorbidity	*“But the unpredictability of caring for people with opioid use disorders and opioid-related health harms, and the fact that they otherwise aren’t engaging on average as much for their physical health needs, we do suddenly find widely metastatic cancers, huge infectious complications that we’re prepared as a team and having those conversations with people that, ‘Okay, you’re doing okay now, but we may suddenly have to switch.’”* (Physician, home/hospital/shelter)
	*“But with the patients, it is that they are very challenging to engage. They’re often—there’s a lot of psychosocial vulnerability. They’re often desperate for care. They want the support. But they are a very high needs population. They miss more than half of their appointments. They’re very difficult to reach by telephone to reschedule appointments. They’re not the most reliable patients. And I always worry that they will fall through the gaps.”* (Physician, hospital)
	*“I think a lot of the deaths are happening because of kind of missed and untreated chronic diseases. I find that there’s, in general, we have a lot of liver failure. There’s a lot of people that end up having cirrhosis of the liver and end-stage kind of liver failure. That and COPD. COPD is a huge one as well, and a lot of complications that come from that, probably because of being outside and being exposed to different elements.”* (Paramedic, community)
	*“Well, ‘This person could get potential life-saving therapy if they could go to rehab.’ And it’s like, ‘Well, considering right now she has a palliative diagnosis, she doesn’t have anywhere to live, she manages substance use disorder, she has four kids, she has a history of trauma, like really you think that rehab, we should push that?’ …it’s like asking somebody who’s in their last days of life with lung cancer who still smokes if they’d like some tobacco cessation products. Like, it’s not totally appropriate.”* (Indigenous patient navigator, hospital/home/clinic)
Resiliency	*“There are some people that they look like they’re dying and then you feed them and care for them and they get better and they don’t die because they finally got regular nutrition and pain management and management of infections and opioid agonist therapy on a consistent basis and social support, and often they surprise you by living much, much longer than expected*. *The prognostication is very difficult in these patients. It’s incredible what they can tolerate.”* (Physician, clinic)
	*“I’ve seen people go in that weren’t expected to be around long and they end up in hospice and they’re here a year later. And it’s because the care they got was just, poof. And then they did really well for a long time because of that extra care. And I’ve seen it, so I’m a believer. […] You know, you’ll see like improvement in so many things. And sure, they still have the illness that’s going to take their life eventually. But you see so much improvement in quality of life until that time. And I think that we even prolong that because now they’re in a place that’s conducive to healing, right?”* (Caregiver)
	*“A lot of it is closer to the end of life, but not that it surprises me, but the amount of resiliency in our community has been really amazing. And people can really fool you. When you think that they’re on their way out, and they’re not quite in agreement with that. [*…*] And it’s great to see, too. There’s so much in this work that’s hard to accept, and there’s sometimes very little positive. And it’s really nice when you can see someone cheating death and be able to experience the things in life which make it so joyful—like reconnecting with family, and engaging in their art, or whatever their hobbies might have been, or hiking or music. It’s a nice gift.”* (Harm reduction worker, home/supportive housing/hospice)

Theme 2: Stigma/discrimination in care	
Sub-themes	Quotes
Structural	*“The clients with opioid use disorder have a tendency not to necessarily come, so there is a lot of stigma, I think, around the hospice.”* (Nurse, community-based hospice)
	*“A palliative approach in a controlled, community-based setting like a hospice or…supportive housing keeps people out of hospital and out of the ER [emergency room]. It’s not talked about enough. It’s often frowned upon a little bit, like taking patients with serious illnesses who are more upstream from end of life and treating their symptoms and pain in a way that you might treat someone who’s later in life, but actually, it keeps them happy, keeps them out of the ER, and it actually prevents hospitalizations. I wish that would be celebrated a little bit more. It tends to be stigmatized in our healthcare discourse a little bit. […] I think we as a palliative care community really need to de-stigmatize the work we do. And, you know, given that opioid use disorder is causing so much more morbidity and mortality for folks, why do we treat this condition differently than we do cancer, or kidney failure, or any other serious life-limiting illness when it is itself a serious life-limiting illness?”* (Physician, hospital/home/clinic)
Social	*“One bad encounter can really impact their comfort level and their ability to access care. So even if it’s not stigma from the provider with ongoing active care, it could be something happened in the past that’s influenced their ability to trust their healthcare team.”* (Physician, hospital/home/palliative care unit)
	*“They’re often labeled as difficult. And sometimes they’re labeled as difficult because they are not feeling heard.”* (Physician, hospital/clinic)
	*“They called up my family…and told them I was full of track marks everywhere, and I only had one. To this day, I only have one track mark, and it’s not long. They told my family I had track marks from head to toe, everywhere.”* (Patient)
Self	*“I just know so many people feel so much shame and so much guilt that they won’t go to a needle and syringe program. They won’t go to an overdose prevention site. And it’s a sin. Because even just contracting hepatitis C, if you have hepatitis C for a long time and you don’t get it treated, you could die from it, right? I know a lot of people that have died from hep C, and I think that stigma is a huge role, right?”* (Patient)

Theme 3: Broken healthcare systems	
Sub-themes	Quotes
Lack of resources	*“I know simply, you think it’s just, ‘Here’s your medications. Here’s your side effects. Here’s what to expect.’ But we end up, due to the, I guess I would say, lack of mental healthcare available and as well with the lack of family medicine available to people, we end up being that main education and that hub.”* (Pharmacist, home/pharmacy)
Restrictive policies	*“You just don’t want them to have to modify their use if they don’t want to do that just because they need institutional care. Does that make sense? But sometimes you have to put in boundaries. You can’t use cocaine on the palliative care unit. It’s illegal.”* (Physician, hospital/home/palliative care unit)
	*“And then other things for patients who are admitted to hospital and they go out on pass and they come back intoxicated. That sometimes happens as well. And that causes a lot of distress on hospital units and that sort of thing who are not sure how to manage those patients.”* (Physician, hospital)
Lack of provider training/knowledge about addiction	*“That is perhaps a gap in the education that we’re providing in the course of our training. And I just wonder if we did get more exposure and more formalized training, whether there might be an increased degree of comfort in caring for patients with opioid use disorder. I think within my own group, there’s a handful of us, and the consults are shunted off to us because people know that we’re perhaps more familiar, more comfortable in caring for these patients. But then that means that our colleagues don’t get the exposure, and I think if we could start early on maybe and do training early, then perhaps there would just be increased comfort across the board.”* (Physician, hospital)
	*“Some of the admitting to the surgeons and stuff are not explaining that very well because they’re uncomfortable having that conversation, and they’re uncomfortable talking to people who use drugs, and they’re afraid some people are going to get angry. They’re afraid of, who knows. But they will often have to go in afterwards and be like, this actually means that antibiotics are only going to work so much.”* (Nurse, hospital)
	*“I think the barrier is actually finding a clinician who’ll care for them, and then once they do, I think it’s a barrier in the sense that when they do access traditional palliative care services, like home care, for example, I’m not convinced that all our colleagues are well-versed or understand how things are different for this population.”* (Physician, hospital/home/clinic/street/shelter)
Lack of information about palliative care	“…*I’m cancer-free. I don’t know why—I still don’t know why I ended up in palliative care. I don’t know who referred me there. I have no idea. I just know that once I got that blood infection, they said I had an appointment at palliative care. I didn’t even know what palliative care was until I went.”* (Patient)
	*“All I knew about her and the palliative care is that she was going to be the doctor that gives me my pain pill.”* (Patient)

Theme 4: Interdisciplinary care	
Sub-themes	Quotes
Within institutions	*“There’s no formalized network, although I will say, it is something that we have received a little bit of funding to work on. So, we’re trying to develop sort of a harm reduction resource team for the hospital so that there is something that’s a little bit more formal where people can turn if they require additional supports beyond just what can be obtained through the addiction medicine service.”* (Physician, hospital)
	*“At the hospital site I’m at, more recently there is a psychiatric team that’s geared towards helping folks with addiction, and so making sure that that team can be involved, given their expertise. I’m not an expert on trauma-informed care, so making sure that I have social work and any interprofessional provider that’s available that could help. I think a team-based, interdisciplinary and interprofessional approach is the one I would take. It’s not always available, which is frustrating.”* (Physician, hospital)
Between institutions and community settings	*“I think having access to teams like the [palliative care outreach] team, the [other palliative care outreach] team, in every community, not just the fortunate ones that are like the big centers with one person who really has been influential in getting that up and running and maintaining it and continuing it on, but that it be sort of a universal, something that’s available in all communities just because it is—I’ve just noticed how much value it has for patients in terms of establishing those long-term relationships and that trust. That is so hard to do when you only meet them for a few days or a short time in hospital. And so that team to be able to be there in the community, as well as to provide some support in hospital as well, I think is truly amazing.”* (Physician, hospital)
	*“I connect. Because doing navigation, I’m the common thread that tries to connect everything together because healthcare, everybody works in such silos and don’t talk to each other. So, my whole role is to try to create some of that continuity and make sure that everybody is on the same page, especially with our folks who have so many complex symptoms. And so much—like, the social determinants of health are complex to navigate. And again, our folks need that completely holistic approach.”* (Indigenous patient navigator, hospital/home/clinic)

Theme 5: Tailored and innovative approaches	
Sub-themes	Quotes
Patient-centered, trauma-informed care	*“Number one is to build rapport and gain some trust if possible because a lot of people, especially our population, require more, I guess, patience and time to take care of them because they’ve gone through a lot of different systems that have let them down. And you have to make them comfortable enough for us to care for them. So, at the beginning is always getting to know each other, to build trust.”* (Nurse, community-based hospice)
	*“I think the strategy for this population is really having that flexibility for your care.”* (Palliative care clinical coach, First Nations community)
	*“To me the priority is alleviating suffering, it’s not reducing addiction or overdose risk. That to me, and that’s the same feeling I get from a lot of people who’ve been through this – is they’re not afraid of dying, they’re afraid of being in pain when they die.”* (Harm reduction worker, home/supportive housing/hospice)
Harm reduction	*“Even when talking about using a harm reduction approach, I’ll say, you can go to this place, and you can smoke, you can drink, you can do whatever you want to do—you do you—and I just try to create that safe environment so that the client can, if they feel that they want to, can be honest and say what they actually are doing with their lives in an attempt to meet them where they’re at.”* (Indigenous patient navigator, hospital/clinic/home)
	*“Just thinking about the people that I’ve been involved with that we’re trying to give good palliative care to, but the mainstream system often makes it really challenging for those people to get good palliative care. And it’s been something over the years I’ve been really proud to be part of, where we have found alternative ways of getting people care at the hospice, where they’re injecting in our hospice.”* (Nurse, community-based hospice)
Community-based supports	*“The best thing you can do for anybody is shelter. It’s like Maslow hierarchy or whatever it is. Shelter can stop people from going to the hospital. If they have a roof over their head, it’s stabilizing people a lot. It works more than giving somebody a psychotic. If somebody has a roof over their head that they can call their home and have food on their table that they can eat and they don’t have to think about where they’re going to get their next meal, it solves more than half of the problem.” *(Nurse, congregate living)

### Complex health and social needs complicate prognosis and illness trajectories

Structural vulnerability framed the end-of-life experiences of many people with OUD, primarily poverty and homelessness, which impeded access to healthcare while worsening health:

*“There’s a lot of 40-year-olds who I would not be surprised if they died for a non-overdose reason. I think of them as dying of old age in their forties, as a consequence of the chronic use and the homelessness and the exposures their bodies have had.”* (Physician, clinic)

A lack of family caregivers was also common and affected access to care for life-limiting illness:

*“Our services are set up for families, I find. Like, it’s even the language is families. As soon as you have cancer, especially, it’s all about your family and what services are going to involve. And I think that’s really difficult…I just think that that alone is a barrier—is to assume that you have someone else to help you make decisions, to go home and talk over decisions. And that’s not necessarily the case.”* (Nurse, clinic/community)

Many people in this community were focused on meeting their basic needs, prioritizing shelter, food, substance use and safety, which involved constantly searching for resources and transportation. Participants called this “survival mode” and described how it permeated their lives: *“I just don’t want to die, right? Like, everything that I do seems like it’s all about trying to stay alive. And it’s a constant struggle to stay alive.”* (Patient).

Participants noted that this common experience meant that future-thinking activities, like treatment plans or advance care planning, *“…can’t even compete with their current priorities of today.”* (Nurse, clinic/community).

Moreover, many people with OUD experienced long-term marginalization from mainstream society, resulting in challenges navigating healthcare systems:

*“In this palliative space, because there's no way we can support folks that have been marginalized for so long and then push them into a cancer system, right, and expect them to show up for their appointments and things to go well for them. The outcome won't be great unless they get the supports.”* (Caregiver)

Relatedly, people with OUD highly valued their autonomy and feared they would lose control over their drug supply or be socially isolated from their community if they entered institutional care:

*“He just didn't want to go in because he wanted to be around the people, his community, people he trusted. He was actively dealing, and how would we do that if he was at a hospice? He can’t. And then he doesn't have money*… *And the cycle goes on. So, I think they often will want to be in their own setting. And then they will often have a critical event that brings them into a hospital, unfortunately.”* (Physician, hospital/home/palliative care unit)

From a provider perspective, patients’ complex health profiles, including physical, mental, and substance use disorders, meant that mainstream services were inaccessible or did not meet their needs: *“Like, profound mental health challenges that don’t fit into that nice norm box of what a lot of the, you know, traditional healthcare or palliative care centers/places, like, require and accept*…*”* (Nurse, community-based clinic).

Patients’ extensive multimorbidity also meant that providers perceived their deaths as more preventable and related to the progression of untreated or unmanaged chronic illness. This lack of engagement in care meant that patients often presented late and had missed diagnoses. Palliative care was often started late and of limited quality, and patients advanced quickly to death. Hence, negative end-of-life experiences were common, including admittance to hospital very near to death:

***“*** *Sometimes we've been involved, and the patient just is so close to death, to be honest, that it's happening within a week of them being admitted. […] because they come to the hospital so ill already. We're like, this person is not going to make it out of the hospital, especially when it's, I would say, the polysubstance, like the alcohol and the opioids*…*”* (Nurse, hospital)

Furthermore, identifying when people with OUD became “palliative” was challenging and some received a palliative approach to care throughout their chronic illness trajectory:

*“…in the clients that we serve, I find that, if you talk about end of life or death, that they spend a lot of their time not thinking about it or wanting to talk about it. And so, what we consider palliative care is sometimes different from what traditionally would be on that scale.”* (Nurse, clinic/community)

Due to this complexity, providers noted reluctance to care for these patients and a tendency to offload them to other providers: “*It’s hot potato, nobody wants these people.”* (Physician, hospital/clinic). As such, patients also received lower quality care early in their serious illness trajectory. Providers’ preoccupation with, and the healthcare system’s inability to accommodate, substance use in treatment protocols or palliative settings limited patients’ options for care:

*“…lots of the patients are just kind of almost considered semi-palliative just by nature of severe substance use, that, what are we doing with this? They're probably not going to stop using drugs…so are we going to offer them even anything? Which is weird to me, because if you ask people, they're like, ‘Oh, I'd like to live.’”* (Nurse, hospital)

Additionally, providers noted that patients’ life experiences had made them highly resilient, which contributed to difficulty in predicting their disease trajectory and remaining lifespan, and the provision of palliative care sometimes improved their quality of life much more than expected:

*“I have seen a couple of clients passing this year, and it presents differently than traditional palliative care. Our clients are excessively resilient. And so, they can be walking and talking with crazy diseases that you would see the traditional person lying down in bed and dying, and then the next day [they die]—it's very quick sometimes how they present.”* (Nurse, community-based hospice)

### Multi-level stigma and discrimination in care leads to poor outcomes

Participants experienced intersectional stigma and discrimination (i.e., relating to drug use and other factors, like homelessness) at multiple levels (structural, interpersonal, and self-stigma). Structural stigma was most evident in hospitals and often related to zero-tolerance policies or lack of institutional support for harm reduction or even for existing standards of care for substance use disorders:

*“The idea of making someone comfortable enough to come into the hospital for treatment would be the thing that would need to happen, which has only been a few times people contacted me, like outpatient social workers, something like that, about what can you give this patient when they come in, and it just doesn't work out very well, you know? Because I can't assure that no one's going to be an asshole to them in the emerg [emergency department]. It's [a] systemic issue. […] So, it's not really like a team problem, but a whole hospital culture problem of this not being a welcoming place for people who use any kind of drugs.”* (Nurse, hospital)

Structural stigma was also evident in how providers identified OUD or opioid-related risks in palliative care settings. Most did not use standard criteria to identify or diagnose OUD:

*“It’s one of those things that probably gets disproportionately carried forward in charts and warped in a broken telephone kind of manner… It's very rare that anyone in their past medical history has opioid use disorder listed as a formal diagnosis.”* (Physician, hospital/home/clinic)

Charts were the most common way to identify risks, but labels in charts followed patients through subsequent interactions with the healthcare system, encouraging stigma in care:

*“…if it's past IV drug use, it's written in charts. And often, even if it's been 20 years…it's still flagged. And violent behaviors. So then sometimes we’ve seen, unfortunately, full medical teams treating the client like they were an active IV drug user or actively in withdrawal when those clients were not using substances.”* (Nurse, community-based hospice)

Some providers used opioid screening tools to identify risks, while others considered these stigmatizing:

*“We don't use opioid screening tools. We don't believe in them. We find them to be very stigmatizing. They perpetuate stereotypes. We're not the police; we're doctors, we’re healthcare workers. We're not here to police people's opioid use. If you really believe in harm reduction, then you wouldn't need to use opioid screening tools. And I think in a palliative care context, it's especially emphasizing the lack of humanity.”* (Physician, hospital/home/clinic/street/shelter)

Structural and interpersonal stigma often overlapped, particularly in how staff enacted hospital policies, resulting in patients feeling mistreated: *“It’s hell, our medical system. At least for me as a person who uses drugs, more often has ended in a security guard coming to my room than it has with a doctor and follow-up and specialists.”* (Patient/caregiver).

Participants also described how past stigmatizing interpersonal encounters impeded access to care. Providers noted that prior negative experiences were common and led patients to fear institutional settings:

*“All of them have—exactly—institutional trauma. And a lot of them have spent time in jail… mental health institutions, long-term care stays in hospitals and things like that. And, like, we have clients that just—like that is their worst, worst fear. …and they haven't had a lot of control over their lives.”* (Social worker, community-based clinic)

Patients corroborated the effect of cumulative stigma from past encounters leading to avoidance or leaving hospital without care when facing discrimination:

*“I didn't have a fight left in me, and I didn't want my partner to get upset because then they would have a reason in their history of dealing with us for treating us badly, right? If he got aggressive, they would write that he got aggressive or he became rude. He became a threat to other patients' safety. And I've been through that before, and it's like you even raise your voice to match their voice, and all of a sudden, you're violent and aggressive because you're a drug user. So, I calmly just said, ‘They're not going to help us. We have to go. There's nothing we can do here.’”* (Patient/caregiver)

Self-stigma also had a substantial impact on end-of-life experiences among people with OUD: *“I did some drugs. First time in seven months. I’m so ashamed of this. […] I could jeopardize the relationship with my grandson to have a pop of cocaine.”* (Patient).

Consequently, providers highlighted that some patients required outreach because they felt undeserving of care and would not seek it:

*“I really think it's a worthiness thing, and I think it’s so many of our clients who have been to jail, who have done bad things, at the end of their life, are filled with regret and think that karma—that they deserve pain.”* (Nurse, community-based hospice)

### Broken healthcare systems hinder access to quality palliative care

Participants described how the healthcare system’s ‘one-size-fits-all’ structure lacked flexibility to provide the low-barrier supports required to accommodate the complex health and social needs of people with OUD:

*“The system is not set up to have people who miss appointments, who don’t wait well in waiting rooms, who can't get to places, generally, and then, on time, who need support to get there, who have no phone to book appointments—all of those kind of logistical problems.”* (Nurse, clinic/community)

Participants also described the negative effects of siloed care, including poor care coordination and lack of communication across services: *“There is often a lot of siloing, so people have to go here for your food, here for your social assistance, here for your cancer care, here for your palliative care, here for your primary care.”* (Physician, hospital/home/clinic/street/shelter).

Relatedly, participants commonly discussed resource limitations for supporting people with OUD (e.g., funding, staffing), especially with respect to mental health, substance use and social services, sometimes leading other healthcare roles to fill gaps:

*“The amount of need versus the amount of service that can be provided here is wildly out of whack. So, the nurses and the providers end up doing a lot of the social service stuff, too, because*…*that’s who the client is able to open up to.”* (Social worker, community-based clinic)

Furthermore, people with OUD often lacked regular primary care providers, which was considered a critical barrier to accessing palliative care. Limited primary care related to unaddressed healthcare needs, low continuity in care, and more difficulty receiving referrals. Consequently, other providers (e.g., palliative specialists, pharmacists) sometimes delivered care focused on people’s general health needs:

*“These folks often don't have a family doctor who can help navigate the rest of their healthcare issues. So, we often end up having to take that on and with the understanding that I'm not a family doctor. […] And so how do we sort of have that risk discussion of what am I not doing for you from a preventative health perspective, but to try to help navigate those key pieces.”* (Physician, hospital/home)

Another barrier involved the lack of system-level support and the presence of restrictive policies in inpatient palliative settings. Providers noted that zero-tolerance policies were a greater concern for individuals with polysubstance versus only opioid use:

*“Sometimes there is resistance to go to hospice because, again, there are more rules and regulations in terms of what supports or what can be provided from an opioid use and other substance management. Often the opioid part is not the problem. It's often other substances, right? It’s alcohol or other things like that.”* (Physician, hospital)

Lack of addiction medicine training was also a systemic barrier that led to discomfort caring for people with OUD: *“One of the barriers there would be lack of education and training for clinicians, and lack of system and hospital-level supports that would help us and support us to safely and competently provide care for these people.”* (Physician, hospital).

Another systemic barrier was the lack of knowledge about palliative care. Patients/caregivers not receiving palliative care believed it was only intended for people at the very end of life; whereas patients who received palliative care thought it should be promoted or explained better: *“There has to be more information out there. There’s not enough communication about palliative care*…*”* (Patient).

### Interdisciplinary care within and across settings

Collaboration between different types of providers occurred within and between institutional and community settings. Palliative care for people with OUD, or a palliative approach to care, was provided across diverse settings, often outside traditional services by non-specialist providers. Interdisciplinary collaboration was regarded as essential to meeting patient needs, but in practice it was often informal, fragmented or inconsistent*: “*…*we do have an addiction medicine team. It’s sort of chronically underfunded and understaffed, and so certainly we would love for them to have a greater presence, and we do collaborate when we can*…*”* (Physician, hospital).

Within hospital settings, palliative providers discussed utilizing psychiatric or addiction supports, though such services were often unavailable and they specifically desired increased access to psychological and social services:

*“Even though it's a low-frequency population, I think a coordinated, specialized, multidisciplinary team that includes social work, psychiatry, addictions medicine, oncology, and palliative care, that functions in a collaborative care model of care, where there are regular team meetings and there's regular monitoring of outcomes on the patient, would be the best model of care.”* (Physician, hospital)

With respect to collaboration across institutional and community settings, inpatient providers who managed to engage with community-based addiction/harm reduction providers praised the support they received:

*“In the end, we connected with this individual's addiction specialist in the community, who really provided us a lot of guidance about how to manage it in hospital. And so…I personally felt comfortable taking over the prescribing and titration in hospital. And because this patient's specialist was so wonderful, we were able to successfully keep them in hospital through their treatment and then transition them back to her care when they left. So, I would say the key piece there was the collaboration and the incredible support from the community team.”* (Physician, hospital)

Accordingly, providers with OUD expertise described the need to advocate for their patients navigating traditional care settings, sometimes through formal patient navigation but more often by pushing the boundaries of their roles:

*“A lot of times when I have to refer patients to the ER [emergency room], I'll write my cell phone number on the card and say, have the doctor call me. We're only open till 2:00. I know you might be looking at a three-, four-hour or day wait to get through emerg, but don't screw up with that number. I'll block you. But I was like, tell the doctor to call me because as soon as they see your history, they're going to have questions and probably give you a hard time for treatment.”* (Pharmacist, home/pharmacy)

### Tailored care approaches and innovative strategies

Providers with OUD expertise offered a variety of unique, targeted strategies in caring for people with OUD and life-limiting illness, all reflecting a philosophy of “meeting people where they’re at”.

#### Providing patient-centered, trauma-informed care

Providers explained that patient-centered care for this community required trauma-informed approaches:

*“…most of the principles of a trauma-informed approach resonate with me. […] there's just a gulf of despair and mistrust that has to be bridged before someone with an opioid use disorder and serious illness can give you a reliable account of their symptoms that you can both believe in.”* (Physician, hospital/community-based hospice)

Accordingly, providers highlighted the importance of incorporating flexible, holistic approaches that fostered trusting and respectful relationships:

*“Because we build such good rapport, people mostly tell us up front… And, you know, it's a very casual kind of approach, and people tend to open up when you're just human with them, real with them, right?”* (Physician, hospital/home/clinic/street/shelter)

One patient receiving palliative care in a community-based hospice corroborated the importance of trust and described a more beneficial experience than anticipated:

*“They're like my family. Like, [unit nurse], there's nobody I trust more than her. Like, the first two months I was here, I couldn't have any staff help me shower. I'm very modest. And a couple of days ago, she gave me a shower. And that's a big step for me, a big step.”* (Patient)

Some providers also described implementing specific measures that improved accessibility for patients with mental or physical disabilities:

*“I had one patient. He's passed away now, but basically, my secretary knew that she would need to call him an hour before his appointment with me when he came up. Get out of bed. Call yourself a taxi and get to the hospital. You have your appointment with the doctor. And when she did that, he would be here every time. But if we didn't do that, if we just expected him to remember the appointment we gave him a month ago or two weeks ago or one week ago, inevitably he would still be asleep and not come.”* (Physician, hospital/home)

In addition, participants desired more culturally safe supports, with several noting the distinct needs of Indigenous patients:

*“…there is specific populations that are more marginalized and harder to access…including racialized communities. […] We have an Indigenous healer that's available, but I think that's a whole other thing that is a struggle of trying to meet, especially this population.”* (Nurse, community-based hospice)

#### Centering harm reduction approaches

Providers expressed the importance of incorporating harm reduction strategies into care for this community, like supporting safer injection practices, and they felt that harm reduction and palliative care were based on similar principles:

*“We can offer using drugs outside, like escorting them to make sure that everything they can do*. *Because harm reduction and palliative care is actually the same philosophy, when you take two seconds and you think about it, it's focused on the client's need.”* (Nurse, community-based hospice)

Providers also explained how offering tailored harm reduction supports resulted in immense gratitude, which they noted was due to patients lacking prior positive experiences where providers met their needs:

*“The specific forms of opioids that we're able to give people…the amount of suffering that you can relieve, and the amount of gratitude that you can get from that is very unique. [*…*] sometimes when they hear that you're going to prescribe opioids for their physical dependence, their opioid use disorder, in a manner that is meeting them where they're at, sometimes people cry. Sometimes people hug you. Sometimes people tell you that you're the best healthcare provider, that it seems too good to be true.”* (Physician, hospital/home/shelter)

#### Bringing services into communities

Providers and patients/caregivers alike highlighted mobile outreach teams as a highly impactful service for people with OUD and life-limiting illness:

*“I think the most important—I said I had these outreach nurses that do mobile outreach street health. [*…*] And they're the ones that I really felt comfortable talking to about my leg, showing my leg. They taught me how to clean it, how to dress it, how to take care of it. They helped me find other veins to inject in, so I wasn't further damaging that wound. And that was just a game changer for me. I really feel like if I didn't have that care, I would have lost my leg. If I lost my leg, I could have died*…*”* (Patient)

In addition, patients/caregivers highlighted the importance of peer workers (i.e., support workers with lived expertise often employed in community-based substance use services) and the need to increase these services, including to ensure patients without family/friend caregivers have advocates:

*“We're not promoting enough peers, so when the people that are being paid to do outreach go home at 4:00, there's still people out there. There should always be people out there. And they should be people that are out there anyway, right? We should be supporting those people, those peers.”* (Caregiver)

Some providers noted the value of integrating palliative care with supportive housing, with the need for more units permitting substance use:

*“*…*lots of housing, especially supportive housing, doesn't allow substance use. So, our housing is specialized, in that we expect and rejoice in people who use drugs. […] there's been a long time where there was literally no place for people with health-related harms from opiate use to live where they could get the care they need.”* (Nurse, community-based hospice)

Providers also noted that isolated patients often listed them as next of kin, and they offered a variety of post-mortem services not available in traditional palliative settings:

*“One thing that also is really important that we do in palliative care with our clients that have opioid use disorder or any addiction is actually you take care of all the post mortem that a regular hospice never does. So, we do memorials. We claim bodies so people die with dignity. And we even have access to a cemetery plot if there's no family, because that's a big issue is people were dying and staying in morgues. That's not offered anywhere else*…*”* (Nurse, community-based hospice)

Finally, some patients described their preferences for future care, which centered on community-based approaches. Many hoped to avoid inpatient settings, especially hospitals, and one described a more informal community-based alternative as their ideal end-of-life setting:

*“A community-based hospice is what I would prefer. I don't think I'd want to be hospitalized for a very long period of time. […] If it was a harm reduction-based, kind of community-based thing in an urban area close to where people sell drugs or where people use drugs, that would be helpful, I think. Having access to family and friends on a constant basis, access to good food, television, electronics, if you could have somebody come sleep over or whatnot. […] I feel like I would have the best chance of having a good end of life if I was in a very communal-based, kind of supportive living environment.”* (Patient)

## Discussion

### Summary of findings

This study highlighted the pervasive barriers that people with OUD face accessing quality palliative or end-of-life care, including complex health and social needs, stigma/discrimination in care, and broken healthcare systems. These challenges led to missed diagnoses, limited palliative care, reluctance to enter inpatient settings, and difficulty with prognostication, resulting in common negative outcomes. Conversely, the few patients with OUD who received specialized palliative care cited mostly positive experiences. Interdisciplinary collaboration was both a barrier due to limited capacity and lack of systematic approaches, and a solution in the creative ways that providers implemented such strategies. Other solutions existed in providers’ philosophy and approach to care, including innovative, tailored supports for this community. These solutions comprised patient-centered, trauma-informed and harm reduction approaches, and many unique community-based services, though these too were under-resourced.

### How does this research fill gaps in the literature?

Most prominent in our data was the extent of healthcare access barriers that people with OUD encountered when facing life-limiting illness. Substance-related stigma is a well-known barrier of healthcare systems,^15,23–26^ as is the fragmentation and resource-scarcity of these systems, and limited primary care among people with substance use disorders.^38^ Most hospitals enforce zero-tolerance policies and lack integrated harm reduction programs, making them stigmatizing environments for people who use drugs, despite recent efforts to integrate harm reduction.^39–41^ Our findings align with prior work among patient groups with overlapping experiences (e.g., other substance use, socioeconomic inequities), demonstrating that people experiencing life-limiting illness and structural vulnerability fear discrimination in palliative care.^14,42^ As a result, if patients with OUD expect stigma, or providers hold stigmatizing views, discussions about palliative care may be delayed or avoided, care may be poorer, and referrals may be fewer.

The multimorbidity and complexity of health and social conditions that people with OUD experienced near the end of life resulted in providers’ difficulty with prognostication. Prior literature reports similar issues among people experiencing structural vulnerability,^11,43^ homelessness,^16,17^ or drug/alcohol use broadly.^13,18^ Our study responds to recommendations to investigate the needs of specific sub-populations within these groups (e.g., people with OUD who are experiencing homelessness).^44^ Given the complex needs and institutional trauma that people with OUD experienced, many of the addiction and harm reduction providers utilized a “palliative approach” to serious illness care.^45^ This approach enabled providers to build rapport and improve patient engagement in serious illness care, thus better supporting people who are reluctant to engage with specialized palliative care services. The approach also allowed providers to better address the non-cancer life-limiting conditions that are common causes of death among these patients.^3,4^ Non-cancer conditions and multimorbidity have less predictable trajectories and additional complexity determining progression from chronic illness to life-limiting illness requiring palliative care.^46^ Stigma and other systemic challenges compound this complexity and clinical uncertainty to affect care trajectories for people with OUD.

Another key element of our findings concerned the need to enhance interdisciplinary collaboration to address the complex needs of people with OUD. Most examples of successful collaboration within or between institutions and community-based organizations were based on informal relationships or temporary or underfunded services. Providers desired more interdisciplinary teams, conforming to recommendations in the literature for other structurally vulnerable groups, which suggest that interdisciplinary, intersectoral, and integrated models should include end-of-life care and social services.^44^ In qualitative investigations of specialist palliative care practices, providers routinely describe interdisciplinary teamwork as fundamental to communication, care planning, and holistic support, while highlighting resource and organizational barriers that impede optimal functioning.^47–49^ As such, research commonly suggests the need for improved training and collaboration across disciplines and sectors. However, our findings coincide with a study on end of life among people with substance use histories in the United Kingdom emphasizing the need to invest in more specific solutions, such as patient navigators.^19^ Other unique models of care should also be considered, like harm reduction-based palliative care clinics for patients with substance use disorders and cancer.^50^ As our participants highlighted, similarities in the philosophies of palliative care and harm reduction can facilitate these inventive models and collaborations.

Due to the extensive challenges with access and collaboration in institutional settings, community-based services developed innovative and tailored palliative approaches to caring for people with OUD and life-limiting illness. Such services – including street outreach and care within shelter or supportive housing settings – have demonstrated promise as an emerging response to structural barriers,^51,52^ but are often unsustainable, small-scale pilots funded by short-term grants, making them difficult to scale, adapt, or rigorously evaluate. Sustaining community-based services that demonstrate success on key metrics (e.g., better symptom control, goal-concordant death) requires stable funding, institutional partnerships, and policies that support harm reduction and wraparound social supports.^53^ These supports are increasingly limited in parts of Canada, including Ontario where much of our study data was collected. For instance, Ontario’s government recently forced the closure of many harm reduction services (e.g., supervised consumption sites), further reducing access to care for people with OUD and life-limiting illness.^54,55^

Finally, a trauma-informed approach – now recommended for palliative care, in general^56,57^ – was critical for this community.^58,59^ Trauma-informed care often requires “low-threshold” services,^60^ such as community-based, outreach-oriented models that adapt care delivery to patients’ social realities. This approach is reflected in shelter-based, mobile, and specialized palliative care services developed for structurally vulnerable groups.^14,51,61,62^ However, novel palliative care models, including peer supports, may be required to better meet the needs of these patients. For example, given patients’ hesitance to enter hospice settings, even those designed for marginalized communities, and their strong preference to remain integrated in their community, a community-driven model of care led by people with lived expertise may better support structurally vulnerable people with OUD.

### Strengths and limitations

While our study had many strengths, in particular the integration of perspectives across patients, caregivers, and healthcare providers who held a wide range of expertise, roles, and practice settings, there were also limitations. Despite recruiting for 18 months, we encountered challenges recruiting patients receiving specialized palliative care, potentially due in part to opioid-related stigma. We did not pilot test our sociodemographic surveys, though they were informed by patient, caregiver and provider partners. There were also discrepancies in definitions of OUD and opioid-related risks between patients and providers, and among different providers, reflecting the diversity of participants and inconsistency in application of these labels. This variability in definitions may relate to our finding that some providers were more focused on opioid-related risks than others and described similar care practices for patients with an active versus a distant history of OUD. Despite such varied perspectives, our study recruited many providers with extensive expertise caring for people with OUD; thus, the commonality of stigma in participant reports cannot be discounted. Finally, our qualitative data often highlighted the experiences of marginalized individuals (e.g., homelessness, isolation), which is frequent among people with OUD, yet does not represent all individuals’ experiences (e.g., higher income, more family support).

## Conclusion

Many people with OUD face complex health and social needs, stigma/discrimination in care, and broken healthcare systems that impede access to quality palliative and end-of-life care. End-of-life outcomes are often negative for this community, particularly reflecting patients’ interactions with hospitals. However, alternative, community-based palliative care models tailored toward these patients are better able to ‘meet people where they’re at’ and support care preferences, despite limited resources. These findings can inform practice change via highlighting the need to improve support for community-based approaches and interdisciplinary teams, as well as remove restrictive policies in hospital and other inpatient settings. Palliative care systems should further embrace and integrate patient-centered care, which includes trauma-informed and harm reduction-oriented approaches for people with OUD, with greater investment in the scale and spread of current innovative community-based services. Improved training and collaboration are also required for mainstream palliative care providers and addiction medicine/harm reduction providers to ensure marginalized patients with OUD receive sufficient care and advocacy across settings.^63^

## Supplemental material

Supplemental material - “It’s hot potato, nobody wants these people”: Palliative and end-of-life experiences among people with opioid use disorder and life-limiting illness Supplemental material for “It’s hot potato, nobody wants these people”: Palliative and end-of-life experiences among people with opioid use disorder and life-limiting illness by Lisa M. Boucher, Jenny Lau, Rebecca Bagnarol, Kyle Drouillard, Ladees Al Hafi, Sheila Jennings, Andrew McLeod, Camilla Zimmermann, Teighan Killackey, Joanna Binch, Anne Meaghen Hagarty, Taliesin Cahill, Nicole Buchanan, Tara Gomes, and Sarina R. Isenberg in Palliative Care and Social Practice.

## Data Availability

De-identified qualitative data that support the findings of this study may be available from the corresponding author upon reasonable request and subject to ethical approval.*
