# Supplementary material for: “It’s hot potato, nobody wants these people”: Palliative and end-of-life experiences among people with opioid use disorder and life-limiting illness
Source: Palliat Care Soc Pract. 2026 Sep 28;20:26323524261492741. doi: 10.1177/26323524261492741 (PMC13769996; doi:10.1177/26323524261492741)
Supplement: Supplementary file 1 [file sj-pdf-1-pcr-10.1177_26323524261492741.pdf]

OUTLOUD study – Demographic Form: People/Caregivers

Participant ID: ___________________________

1. Who is completing this survey?
    - Person with life-threatening illness, or person and caregiver together
    - Caregiver only (participating on behalf of your family member/friend)

*Questions for if Person or person/caregiver together [If Caregiver only, skip to question 11]*

2. What is your age?
    - Under 18
    - 18-44
    - 45-54
    - 55-64
    - 65-74
    - 75-84
    - 85-94
    - 95+
3. Do you live in an urban or rural area?
    - Urban (more than 10,000 people)
    - Rural (less than 10,000 people)
4. What is your gender?
    - Woman
    - Man
    - Non-binary
    - Other (Please specify: ________________)
    - Prefer not to say
5. Are you Indigenous?
    - Yes
    - No
    - Prefer not to say
6. Are you a visible minority?
    - Yes
    - No
    - Prefer not to say
7. Which of the following issues have you experienced in the last 3 years that are related to your opioid use? *(Check all that apply)*
    - Visited emergency department
    - Hospitalized
    - Received opioid agonist therapy (e.g., Methadone, Suboxone)
    - Other (Please specify: ________________)
8. Are you receiving palliative care?
    - Yes

- No

*[If Yes, continue to question 9; If No, skip to last question]*

9. Where have you received palliative care? *(Check all that apply)*
   - Hospital
   - Clinic
   - Home
   - Long-term care
   - Other (Please specify: _______________)
10. What types of healthcare professionals have been involved in providing palliative care to you? *(Check all that apply)*
    - Doctor
    - Nurse
    - Personal support worker
    - Social worker
    - Pharmacist
    - Harm reduction/community worker (could include peer or outreach workers)
    - Therapist
    - Spiritual counselor
    - Physiotherapist
    - Occupational therapist
    - Speech-language pathologist
    - Registered dietitian
    - Other (Please specify: _______________)

*Questions for if Caregiver only [If Person or person/caregiver together, skip to last question]*

11. What is your relationship to the person you provide(d) care for? (i.e., I am the person's _______)
    - Spouse/partner
    - Child
    - Parent
    - Sibling
    - Friend
    - Other (Please specify: _______________)
12. What is your family member/friend's age (or what was their age at death)?
    - Under 18
    - 18-44
    - 45-54
    - 55-64
    - 65-74
    - 75-84
    - 85-94
    - 95+
13. Does/did your family member/friend live in an urban or rural area?
    - Urban (more than 10,000 people)

- Rural (less than 10,000 people)

14. What is your family member/friend's gender?
    - Woman
    - Man
    - Non-binary
    - Other (Please specify: ________________)
    - Prefer not to say
15. Is your family member/friend Indigenous?
    - Yes
    - No
    - Prefer not to say
16. Is your family member/friend a visible minority?
    - Yes
    - No
    - Prefer not to say
17. Which of the following issues did your family member/friend experience in the last 3 years that were related to their opioid use? *(Check all that apply)*
    - Visited emergency department
    - Hospitalized
    - Received opioid agonist therapy (e.g., Methadone, Suboxone)
    - Other (Please specify: ________________)
18. Is/was your family member/friend receiving palliative care?
    - Yes
    - No

*[If Yes, continue to question 19; If No, skip to last question]*

19. Where has your family member/friend received palliative care? *(Check all that apply)*
    - Hospital
    - Clinic
    - Home
    - Long-term care
    - Other (Please specify: ________________)
20. What types of healthcare professionals have been involved in providing palliative care to your family member/friend? *(Check all that apply)*
    - Doctor
    - Nurse
    - Personal support worker
    - Social worker
    - Pharmacist
    - Harm reduction/community worker (could include peer or outreach workers)
    - Therapist
    - Spiritual counselor
    - Physiotherapist
    - Occupational therapist
    - Speech-language pathologist

- o Registered dietitian
- o Other (Please specify: _______________)

*LAST QUESTION*

21. Is there any other information you would like us to know?
    - o ________________________________________________

OUTLOUD study – Demographic Form: Healthcare Professionals

Participant ID: ___________________________

1. What is your gender?
    - Woman
    - Man
    - Non-binary
    - Other (Please specify: ________________)
    - Prefer not to say
2. What is your role?
    - Physician
    - Nurse Practitioner
    - Advanced Practice Nurse
    - Registered Nurse
    - Social Worker
    - Pharmacist
    - Harm reduction/community worker
    - Other (Please specify: ________________)
3. How many years have you been in practice?
    - __________________
4. Where do you provide palliative care? *(Check all that apply)*
    - Hospital
    - Clinic
    - Home
    - Long-term care
    - Other (Please specify: ________________)
5. Approximately what percentage of your practice are patients with opioid-related health harms (i.e., had an opioid-related emergency department visit or hospitalization, or received Opioid Agonist Therapy to treat Opioid Use Disorder)?
    - __________________
6. Do you work in an urban or rural area?
    - Urban (more than 10,000 people)
    - Rural (less than 10,000 people)
7. Do you have training in Palliative Care?
    - Yes (Please specify: ________________)
    - No
8. Is there any other information you would like us to know?
    - ___________________________________________________

*Version: July 10, 2023*
